# Prediction of nested complementary pattern in argon dielectric-barrier discharge at atmospheric pressure

**DOI:** 10.1038/srep16391

**Published:** 2015-11-10

**Authors:** Weiman Jiang, Jing Li, Jie Tang, Yishan Wang, Wei Zhao, Yixiang Duan

**Affiliations:** 1 State Key Laboratory of Transient Optics and Photonics, Xi’an Institute of Optics and Precision Mechanics of CAS, Xi’an 710119, China; 2 Faculty of Mathematics and physics, Huaiyin Institute of Technology, Huaian 223003, China; 3 Research Center of Analytical Instrumentation, Sichuan University, Chengdu 610064, China

## Abstract

A two-dimensional self-consistent fluid model was employed to investigate the spatiotemporal nonlinear behavior in an argon glow-like/Townsend-like dielectric-barrier discharge (DBD) at atmospheric pressure. The discharge is characterized by a major current pulse with a residual one ahead per half cycle of the external voltage. The two current pulses are operated in glow mode, but with Townsend mode between them. Contrasting spatial discharge structures are complementarily presented not only at two current pulses in the same half cycle but also during the discharge in the two adjacent-half cycles, resulting in the formation of a unique nested complementary pattern each cycle. This peculiar behavior mainly lies in the fact that sufficient charged particles are trapped in the gas gap due to the last discharge and able to dominate the subsequent discharge through the “spatial memory effect”. The charge transport regime reveals that this nested complementary pattern is presented only in a limited range of driving frequency.

The self-organized patterns have attracted much attention due to their complexity and diversity, which are commonly presented in many systems of different origins such as chemical^1^, biological^2^, and physical systems^3^,^4^,^5^,^6^,^7^,^8^. Solitary structures, spirals, waves and Turing patterns have been usually observed in the laboratory conditions. The Faraday experiment, granular flows, and systems based on photosensitive chemical reactions under periodic illumination are representative spatially extended and periodically driven dissipative systems used for studying these patterns^9^,^10^,^11^. In the periodic illumination reaction-diffusion system, a sequence of resonance patterns are complementary observed in the two adjacent-half cycles of oscillation, when the ratio of the perturbation frequency to the natural one is set at a certain level. A general model, referred to as the frequency-locked regime, was proposed to explain this phenomenon^10^. As a typical non-equilibrium dissipative system, the dielectric-barrier discharge (DBD) can also provide various nonlinear pattern formations, such as stripe, concentric ring, hexagon, and square patterns, due to the nonlinear charge conduction process in gas and the accumulated charges on dielectric barrier^4^,^5^. A typical example showed that the concentric ring pattern could be a superposition of two ring patterns that are complementarily presented at two adjacent current pulses per half cycle by measuring pattern dynamics and a continuum coupled map model was proposed to describe the pattern formation in DBD^12^,^13^. These observations show that a complementary pattern could be either formed in the same half cycle or in the two adjacent-half cycles of external oscillation perturbations. But till now, there is no experimental observation or numerical simulation shows that a complementary pattern appears both in the same half cycle and in the two adjacent-half cycles, which here is defined as a nested complementary pattern formation.

In this paper, we report the first simulation results that unambiguously demonstrate that there are contrasting spatial discharge structures complementarily presented not only at two adjacent current pulses in the same half cycle but also during the discharge in the two adjacent-half cycles in an argon dielectric-barrier discharge. A nested complementary pattern is observed. This discharge is characterized by a major current pulse with a residual one ahead. The spatial discharge structure in the negative half cycle is just opposite to that in the positive half cycle, although the current pulse is symmetric in the two adjacent-half cycles. This behavior is closely associated with the residual discharge that has been identified both in simulations and in experiments^14^,^15^,^16^. All efforts of this work are to gain insights into this spatiotemporal nonlinear behavior and obtain the physical mechanism behind the nested complementary pattern formation.

## Results

### Voltage-current characteristics

**Figure 1:**
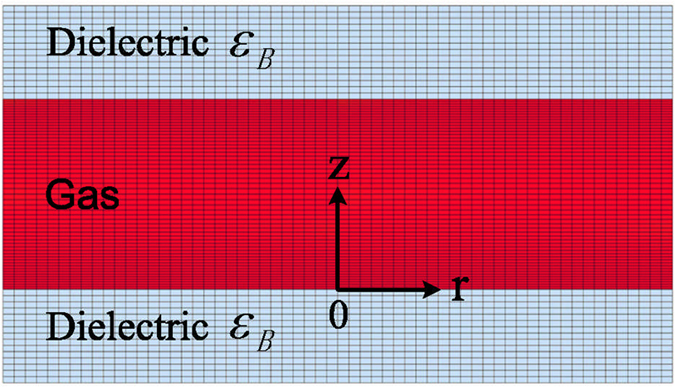
Dielectric-barrier discharge configuration and the simulation domain in an axisymmetric cylindrical coordinate (*r*−*z*) for the atmospheric-pressure argon DBD.

**Figure 2:**
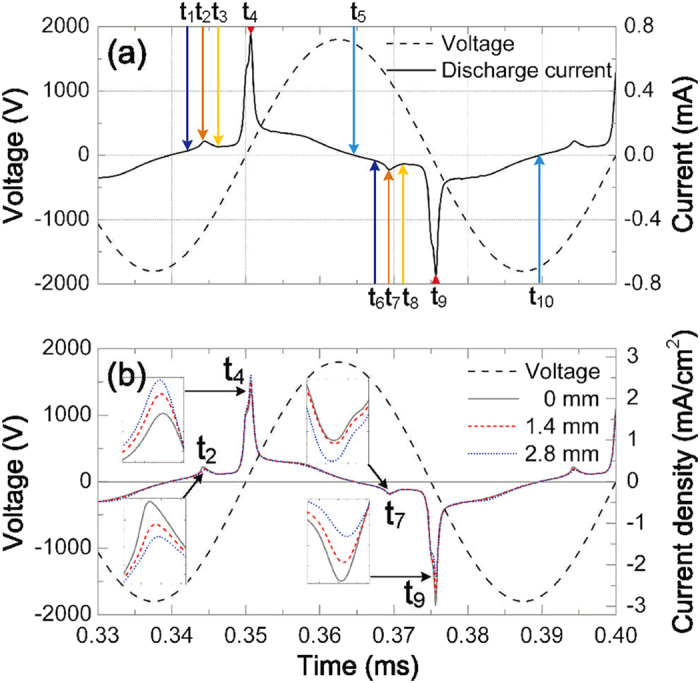
The waveforms of (**a**) the discharge current and (**b**) current density at various radial positions with the radius of 0, 1.4, and 2.8 mm, together with the waveform of the external voltage at the driving frequency of 20 kHz.

Our simulation was carried out based on a two-dimensional (2D) self-consistent fluid model in an axisymmetric cylindrical coordinate (*r*−*z*), as shown in Fig. 1. At the driving frequency of 20 kHz, a typical current waveform over one period, obtained by integrating the current density throughout the electrode surface, is shown in Fig. 2(a), together with the waveform of the external voltage. A major current pulse is always accompanied by a small residual one at the beginning of the discharge per half cycle, because the trapped charged particles in the last discharge flow suddenly when the polarity of field is reversed^15^. In addition, a symmetric characteristic of current pulses is presented in the positive and negative half cycles. It seems likely that the temporal nonlinearity cannot be found from the symmetric current waveform. However, the temporal nonlinearity can be distinguished from the waveforms of the current density at various radial positions, as described below. Figure 2(b) shows the waveforms of the current density at three typical radial positions (r = 0, 1.4, and 2.8 mm), as well as the external voltage, over one period. The current density at r = 2.8 mm presents the strongest at the positive major current peak (*t*_4_ = 350.7 *μ*s) and the weakest at the negative major one (*t*_9_ = 375.6 *μ*s), while it presents the weakest at the positive residual current peak (*t*_2_ = 344.3 *μ*s) and the strongest at the negative residual one (*t*_7_ = 369.4 *μ*s). Compared with the temporal evolution of current density at r = 2.8 mm over one period, the case at the center point (r = 0 mm) is reversed at these four current peaks. The temporal evolutions of current density at these radial positions indicate that the current density appears to be asymmetric in a local discharge region and has different waveforms in the positive and negative half cycles. This indicates that the temporal nonlinearity exists in the discharge. In addition, the distinct current density at different radial positions at a fixed time indicates that the discharge intensity is non-uniformly distributed along the radial direction and the discharge exhibits spatial nonlinearity. These discharge characteristics suggest that temporal and spatial nonlinearities simultaneously exist in the argon DBD. This temporal evolution of current density is quite different from those observed both in simulations and experiments performed by Wang *et al.*^17^ and Mangolini *et al.*^18^. In their works, the current density at different radial positions has different values, but for the same radial position, the current density presents the symmetric waveforms in the positive and negative half cycles, indicating that the discharge has the same spatial structures in the two adjacent-half cycles. Based on the asymmetric waveforms of the current density at these radial positions in our case, what happens to the spatial discharge structure?

### Radial distributions of electron density

**Figure 3:**
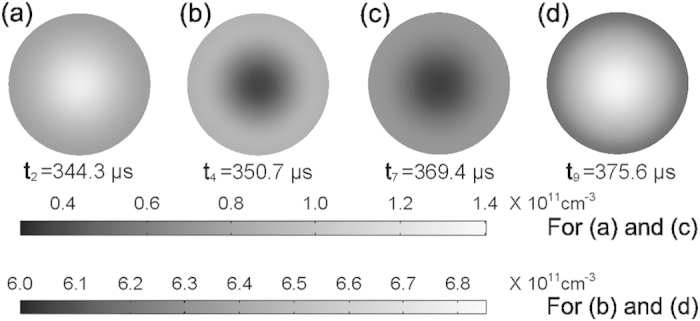
The electron density radial distributions at the residual current peak (**a**,**c**) and major current peak (**b**,**d**) both in the positive (**a**,**b**) and negative (**c**,**d**) half cycles in the plane, where the electron density reaches its maximum. This plane is parallel to the electrodes.

Figure 3 shows the radial distributions of electron density at the residual current peaks and the major current peaks both in the positive and negative half cycles in the plane parallel to the electrodes, where the electron density reaches its maximum. The electron density at the residual current peak (*t*_2_) in the positive half cycle, as shown in Fig. 3(a), has the maximum in the center, declines along the radial direction, and presents an apparently center-advantage distribution. As seen from Fig. 3(b), for the positive major current peak (*t*_4_), the minimum of electron density appears in the center, and the density increases along the radial direction and reaches its maximum in the periphery of the gas gap, showing a periphery-advantage distribution. It is known that the discharge current density, whose spatial distribution can be visualized by the discharge luminance in experiment, produces charged particles in the gas gap^12^,^19^. The magnitude of electron density indirectly reflects the discharge intensity in the gas gap. It is found that in the positive half cycle, a relatively intense discharge moves from the center to the periphery and the discharge intensity distributions are reversed between the two adjacent current pulses. A complementary discharge pattern is presented. It is worth noting that the spatiotemporal evolution of complementary discharge in the positive half cycle is very similar to the experimental findings reported in refs. 12 and 18. Figure 3(c) shows that the electron density at the negative residual current peak (*t*_7_) presents a periphery-advantage distribution, which is similar to the case at the positive major current peak, but contrary to the situation at the positive residual current peak. Subsequently, the electron density manifests itself as a center-advantage distribution at the negative major current peak (*t*_9_), as indicated in Fig. 3(d). In the negative half cycle, a relatively intense discharge moves from the periphery to the center and the reversal of discharge intensity distributions also occurs between the two adjacent current pulses, accompanied by another complementary discharge pattern formation. Further comparison of the electron density distributions at the four current peaks in the positive and negative half cycles indicates that an opposite or complementary evolution of spatial discharge structure is also presented in the two adjacent-half cycles. The evolution of spatial discharge structure over one period clearly shows that a complementary pattern is presented not only in the same half cycle but also in the two adjacent-half cycles, resulting in the formation of a nested complementary pattern.

## Discussion

### Mechanism of the nested complementary pattern formation

**Figure 4:**
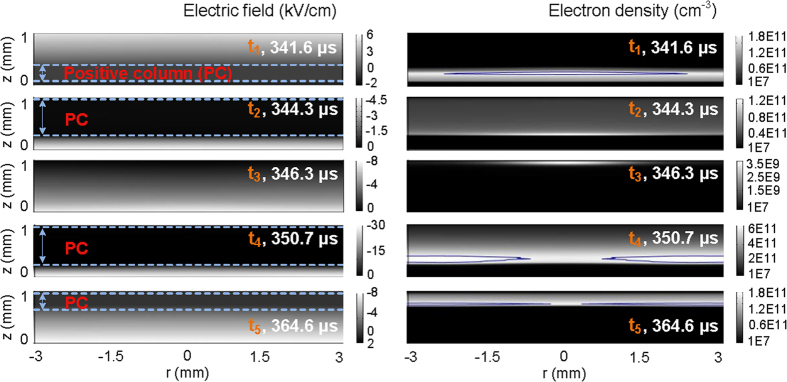
The spatial distributions of electric field and electron density with time scattered in the positive half cycle.

**Figure 5:**
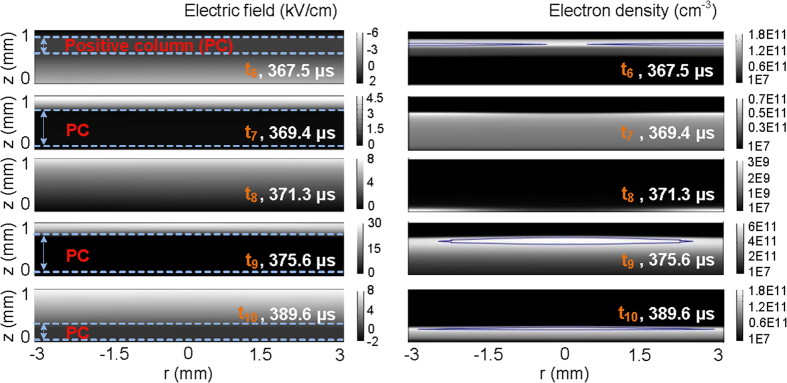
The spatial distributions of electric field and electron density with time scattered in the negative half cycle.

**Figure 6:**
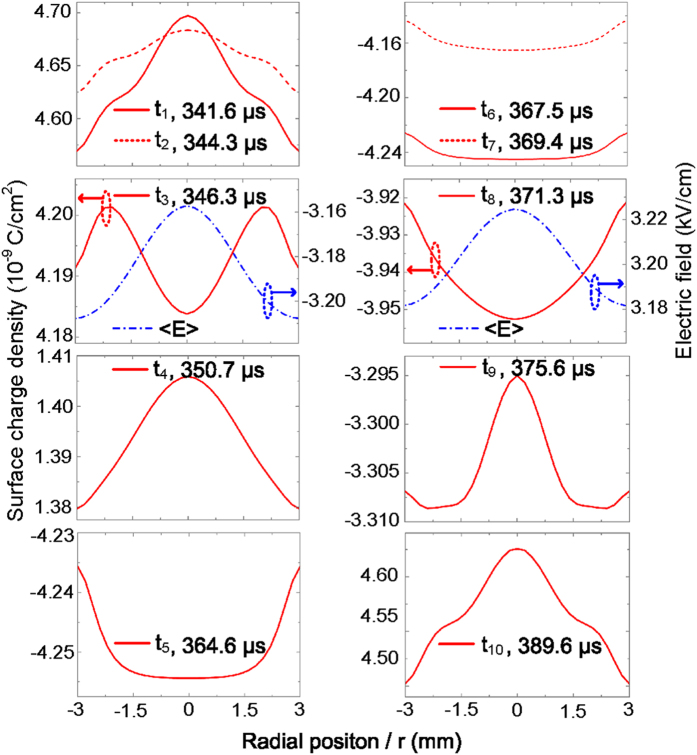
The radial distributions of surface charge density on the upper dielectric barrier with time scattered in one period, as well as the average electric field <E> across the gas gap during the discharge in the Townsend mode.

To demonstrate the formation of nested complementary pattern, the spatial distributions of electric field and electron density are plotted in Figs 4 and 5 with the time increased and scattered in one period (*t*_1_−*t*_10_ marked in Fig. 2(a)). The contour line is added in some subfigures. The electron has a higher number density in the region surrounded by the contour lines than in the area out of the contour lines. Figure 6 shows the radial distributions of surface charge density on the upper dielectric barrier at these scattered moments, as well as the average electric field across the gas gap (defined as <E>) during the discharge in the Townsend mode.

Since space charges trapped in the gas gap are remained in the last discharge and they have no enough time to diffuse and transfer to other places under the low electric field in a very short phase, the similar spatial distribution of charges is maintained as that in the last discharge. This behavior is defined as “spatial memory effect” or “footprints” in the 3-dimentional discharge space, instead of the memory effect on the 2-dimentional dielectric layer for surface charges to be deposited on^20^. At the moment (*t*_1_ = 341.6 *μ*s) before the positive residual current pulse, more charges assemble in the center of the gas gap due to the “spatial memory effect”, as can be seen in Fig. 4. As seed charges, they can initiate a new discharge at a relatively low electric field^15^. Even though the external voltage applied on the upper electrode is still negative, the surface charge density on the upper barrier is positive (the case at *t*_1_ in Fig. 6) and high enough to produce a charge-induced electric field (stronger than the external one) to ignite the residual discharge with the valid assistance of the space charges. At the positive residual current peak, a more intense discharge occurs in the center (the case at *t*_2_ = 344.3 *μ*s in Fig. 4), due to provision of more seed charges there. Therefore, the radial distribution of electron density in Fig. 3(a) is presented. It is found from the case at *t*_2_ in Fig. 4 that the electric field does not vary monotonously from the cathode to the anode and the positive column apparently exists. The discharge sustains the glow mode.

At the time (*t*_3_ = 346.3 *μ*s) after the positive residual current pulse, most of the space charges are exhausted and few new charges are generated in the gas gap due to the low electric field (the case at *t*_3_ in Fig. 4). The maximum of electron density in the gas gap declines to the order of 10^9^ cm^−3^. The discharge turns to be a Townsend mode, which can be concluded from the nearly uniform distribution of electric field in the gas gap. Since the discharge happens more intensely in the center than in the periphery during the positive residual current pulse, more positive surface charges on the central part of the upper dielectric barrier will be recombined with the electrons coming from the gas gap. This results in that the curve of surface charge density sags in the center (the case at *t*_3_ in Fig. 6). This means more positive surface charges are left on the periphery of upper dielectric barrier and a stronger charge-induced electric field exists in the periphery of the gas gap, as indicated by the radial distribution of <E>. <E> means the combined one of charge-induced electric field and external electric field in the gas gap. When the space charge is rare, the discharge would be dominated by the charge-induced electric field under the condition that the external voltage is not so high. Since the ionization coefficient strongly depends on the electric field, the ionization avalanche will begin in the periphery of the gas gap. Thus, a periphery-advantage discharge is subsequently initiated with the external voltage increased. At the positive major current peak, just because of the periphery-advantage discharge, more electrons, as well as more ions, are generated and concentrated in the periphery of the gas gap, as indicated by the case at *t*_4_ = 350.7 *μ*s in Fig. 4. Thus, the periphery-advantage distribution of electron density in Fig. 3(b) is observed. The time-resolved spatial distributions of electron density show that this behavior is maintained until the onset of the negative residual current pulse because of the “spatial memory effect” (the cases at *t*_4_ = 350.7 *μ*s and *t*_5_ = 364.6 *μ*s in Fig. 4, and the cases at *t*_6_ = 367.5 *μ*s and *t*_7_ = 369.4 *μ*s in Fig. 5). Due to the periphery-advantage discharge, more surface charges are neutralized on the periphery of the dielectric barrier, which makes the surface charge density higher on the central part than on the periphery (the case at *t*_4_ in Fig. 6). It follows from the case at *t*_4_ in Fig. 4 that the discharge at the positive current peak is characterized by a glow mode due to the presence of cathode fall and positive column regions in the gas gap. There should be a transition from the glow mode, through the Townsend mode, and back to the glow one between the positive residual current pulse and the positive major one in the same half cycle.

After the positive major current pulse, the polarity of surface charges becomes negative and more charges are stored on the periphery of the dielectric barrier. The increasing surface charges inhibit the discharge through the charge-induced electric field. With the discharge ceased, the accumulation of surface charge on the dielectric barrier is dominated by the displacement current^21^,^22^. The charge transfer to the dielectric barrier is fulfilled mainly through the diffusion mechanism of space charge. The concentration gradient of space charge and the finite aspect ratio of the electrodes give rise to a larger diffusion flux of charge in the center, resulting in assembling more surface charges on the central part of the dielectric barrier^18^, as shown in the case at *t*_5_ and *t*_6_ in Fig. 6. This distribution characteristic of surface charge on the upper dielectric barrier still holds on at the negative residual current peak (the case at *t*_7_ in Fig. 6). Though the electric field is not so high, the remaining space charges with its number density higher in the periphery due to the “spatial memory effect” initiate a periphery-advantage discharge at the negative residual current peak. At the same time, electrons with higher density appear in the periphery (the case at *t*_7_ in Fig. 5). This is responsible for the radial distribution of electron density shown in Fig. 3(c). The discharge at the negative residual current peak maintains the glow mode, which is distinguished from the spatial distribution of electric field at *t*_7_ depicted in Fig. 5. More detailed computational results suggest that the glow mode is always sustained between the positive major current pulse and the negative residual one. The periphery-advantage discharge results in the neutralization of more surface charges on the periphery. As a result, more surface charges are reserved on the central part of the dielectric barrier and a higher charge-induced electric field is formed in the center of the gas gap. As can be seen from the case at *t*_8_ = 371.3 *μ*s in Fig. 5, the space charges are remained few, the cathode fall cannot be formed and the discharge develops into a Townsend mode. Under the condition of rare space charges and low external voltage, the charge-induced electric field plays a key role in initiating a new discharge. <E> with its value higher in the center (the case at *t*_8_ in Fig. 6) initiates a center-advantage discharge at the negative major current pulse with the external voltage increased. The center-advantage discharge makes the space charges dispersed more in the center (the case at *t*_9_ = 375.6 *μ*s in Fig. 5). As a result, the center-advantage distribution of electron density in Fig. 3(d) comes into being. This center-advantage distribution of electron density lasts till the next positive residual current pulse due to the “spatial memory effect”. Since the discharge at the negative major current peak is characterized by glow mode, there should be another transition from the glow mode, through the Townsend mode, and back to the glow one between the negative residual current pulse and the negative major one. More detailed computational results also suggest that the glow mode is sustained until the next positive residual current pulse. After the negative major current pulse, more negative surface charges are neutralized on the central part of the upper dielectric barrier and subsequently more positive surface charges are stored there (the case at *t*_10_ = 389.6 *μ*s in Fig. 6). This distribution characteristic of surface charge is maintained until the next positive residual current pulse. In the next period, the discharge takes the same behavior as stated above.

Based on the interpretation of the discharge process in a whole period, it is found that the evolution of the spatial discharge structure is largely dependent on the surface charge deposited on the dielectric barrier and the space charge remained in the gas gap. The variation of surface charge distribution on the dielectric barrier and the remain of few space charges in the gas gap (after the residual current pulse) give rise to the reverse of spatial discharge structure between the two adjacent current pulses in the same half cycle. The remain of large amounts of space charges in the gas gap (after the major current pulse) allows the discharge at the residual current pulse in the following half cycle to inherit the spatial discharge structure at the major current pulse in the previous half cycle. As a result, a complementary evolution of spatial discharge structure is observed in a whole period, showing us a nested complementary pattern.

### Driving frequency window for the nested complementary pattern

The nested complementary evolution of spatial discharge structure each period is essentially related to the residual discharge. In the phase from the major current pulse to the subsequent residual one, the electron density in the negative glow region, where the electric field almost drops to zero and the electron density reaches its maximum, is gradually reduced mainly due to the diffuse of charged particles. However, discussion above suggests that the glow mode is always sustained between the major current pulse and the subsequent residual one. This means that there still exist a large number of space charges (with the electron density in the order of 10^10^ cm^−3^) in the gas gap, which dominate the spatial discharge structure due to the “spatial memory effect” and allow the residual discharge to maintain the similar spatial discharge structure as that of the previous major discharge. As we know, when the electron density in the gas gap holds at the level of no more than 10^9^ cm^−3^, the electron density increases linearly from the cathode to the anode and the space charge effect is so weak that no cathode fall is formed near the cathode. The Townsend mode appears. In this case, the discharge is dominated by the electric field in the gas gap, instead of the space charge. If the glow mode initiated at the major current pulse is not sustained until the following half cycle and the Townsend mode appears before the onset of discharge in the following half cycle, there should be no chance to initiate the residual discharge for lack of sufficient space charges remained in the gas gap. Therefore, the electron density should be more than the order of 10^9^ cm^−3^ after the decay in this phase from the major current pulse to the subsequent residual one. In addition, the electron density will continue decreasing after the residual discharge mainly due to diffuse when the external voltage is still low. If the electron density still holds at the level of more than the order of 10^9^ cm^−3^ closely before the next major current pulse and no Townsend mode appears, the space charge effect would be great enough to dominate the spatial discharge structure throughout the whole period. The spatial discharge structure will approximately maintain the same in the positive and negative half cycles and no complementary evolution of spatial discharge structure can be found. Thus, the electron density should be reduced to the order of 10^9^ cm^−3^ closely before the next major current pulse.

It is clear that many effects, such as drift, recombination, and diffusion of charged particles, can influence the decay of electron density in the negative glow region. But in practice, it is impossible to take into account all of these effects by an exact method. For this reason, the main effect (diffusion) is only considered and a plane source model is employed to approximately characterize the decay process of electron density in a one-dimensional coordinate. The electron density can be expressed as^23^,


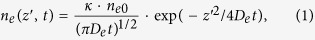


where *κ* is an arbitrary constant, *D*_*e*_ is the diffusion coefficient, and *n*_*e*0_ is the initial electron density just after the major current pulse. The coordinate origin in a non-static coordinate system is considered at the spot where the electron density reaches its maximum in the negative glow region. The electron density at the origin is simplified as


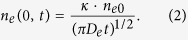


As addressed above, to obtain the nested complementary evolution of spatial discharge structure, there should be a residual discharge in glow mode and a subsequent discharge in Townsend mode before the major current pulse each half cycle. The equation (2) shows that the electron density after the major current pulse largely depends on the decay time *t*, which is closely associated with the driving frequency *f*. When the driving frequency is too low, the interval from the end of discharge in the previous half cycle to the onset of discharge in the following half cycle is so long that overmuch time is available for the electron density to decay to the order of 10^9^ cm^−3^ or even lower. The residual discharge is not initiated due to few space charges remained. On the other hand, when the driving frequency is too high, there is no enough time for the electron density to decay to the order of 10^9^ cm^−3^ before the next major current pulse arrives in the following half cycle. The discharge fails to transform from the glow mode to the Townsend mode before the next major current pulse. In the two cases, the nested complementary pattern is not formed. Thus, the driving frequency is required to be limited in a certain range to observe the nested complementary pattern.

Here, the decay time *t* is written as 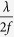 (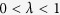). The lower and upper limits of driving frequency can be qualitatively determined from the decay time required for the electron density to decay to the order of 10^9^ cm^−3^ under two typical conditions. When the frequency decreases to the one at which the residual discharge exactly occurs and *n*_*e*_(0, t) falls to the order of 10^9^ cm^−3^ at the decay time *t*_*low*_ soon after the residual discharge, it corresponds to the lower limit of driving frequency *f*_*low*_. When the frequency increases to the one at which the Townsend discharge appears with *n*_*e*_(0, *t*) reduced to the order of 10^9^ cm^−3^ at the decay time *t*_*up*_ closely before the next major current pulse, it is considered to be the upper limit of driving frequency *f*_*up*_. It should be noted that the lower and upper limits of driving frequency cannot be numerically figured out, due to the complexity and incompleteness of the fluid model employed here. Thus, more simulations were carried out at other driving frequencies to roughly determine the driving frequency range. It is found that the nested complementary pattern appears at the driving frequency varied from 17 to 21 kHz. When the driving frequency declines below 16 kHz or rises above 21.5 kHz, the nested complementary pattern cannot be observed. Two extra examples are given below to address this issue.

**Figure 7:**
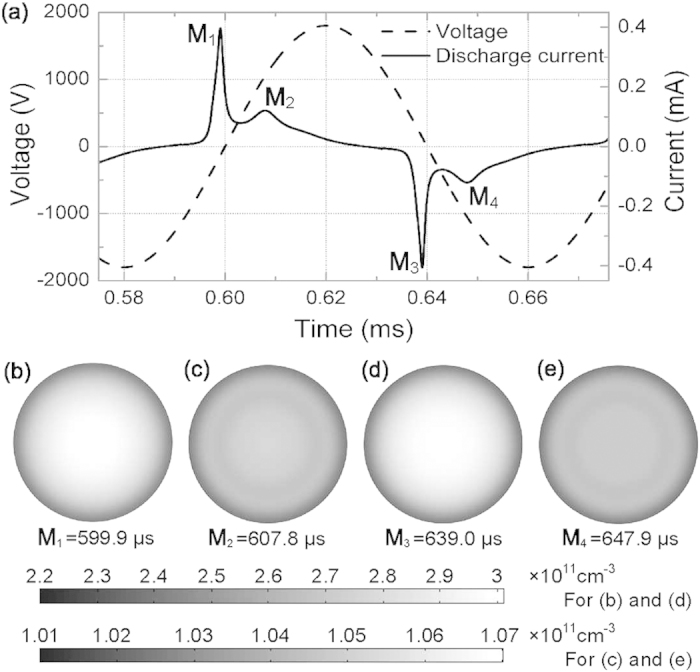
(a) The waveforms of the discharge current and external voltage at the driving frequency of 12.5 kHz; and the corresponding electron density radial distributions at the major current peak (b,d) and second current peak (c,e) both in the positive (b,c) and negative (d,e) half cycles in the plane, where the electron density reaches its maximum . This plane is parallel to the electrodes.

With the driving frequency decreased to 12.5 kHz (less than *f*_*low*_), the waveforms of the external voltage and discharge current over one period are illustrated in Fig. 7(a). A major current pulse, followed by a second small one, is observed per half cycle. But no residual current pulse is found before the major one. This is because there is enough time for electrons generated in the previous discharge to diffuse around and the electron density is reduced to the order of 10^9^ cm^−3^ or even lower before the onset of discharge in the following half cycle. The electron density radial distributions at the major and second current peaks both in the positive and negative half cycles are depicted in Fig. 7(b–e). Here, the electron density is plotted in the plane parallel to the electrodes, where the electron density reaches its maximum. Figure 7(b) shows the electron density radial distribution at the major current peak (*M*_1_ = 599.9 *μ*s) in the positive half cycle, while Fig. 7(d) illustrates the case at the major current peak (*M*_3_ = 639.0 *μ*s) in the negative half cycle. It is found that the electron density has the maximum in the center and drops along the radial direction in both the two major current peaks, where a similar or same center-advantage distribution is observed. As seen from Fig. 7(c,e), the approximate center-advantage distribution of electron density at the second current peak (*M*_2_ = 607.8 *μ*s) in the positive half cycle is quite similar or the same as that at the second current peak (*M*_4_ = 647.9 *μ*s) in the negative half cycle. Here, a concentric ring pattern is indistinctly presented. Further simulations suggest that no Townsend mode appears between the major current pulse and the second one per half cycle. This is responsible for the failure in the formation of a complementary pattern at the two adjacent current pulses in the same half cycle. Comparison of the electron density radial distributions in the positive and negative half cycles indicates that the discharge at the driving frequency of 12.5 kHz has the similar or same spatial structure in the two adjacent-half cycles and no complementary evolution of spatial discharge structure is observed. The nested complementary pattern is not formed.

**Figure 8:**
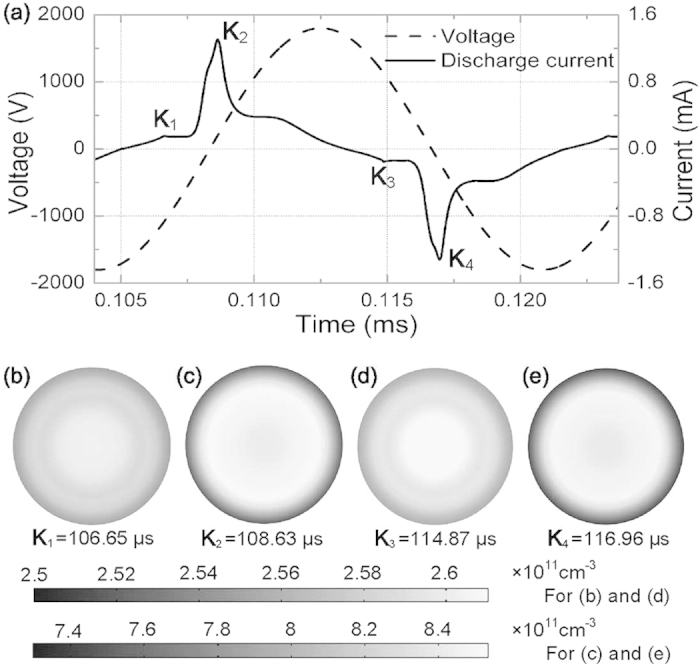
(a) The waveforms of the discharge current and external voltage at the driving frequency of 60 kHz; and the corresponding electron density radial distributions at the major current peak (b,d) and second current peak (c,e) both in the positive (b,c) and negative (d,e) half cycles in the plane, where the electron density reaches its maximum. This plane is parallel to the electrodes.

With the driving frequency increased to 60 kHz, the waveforms of the external voltage and discharge current over one period are shown in Fig. 8(a). A major current pulse is presented with a residual one ahead per half cycle. Further simulations show that there is no Townsend mode between the two current pulses and the glow mode is sustained in the whole period, because there is no enough time for large amounts of electrons generated in the discharge to diffuse around and the electron density maintains above the level of the order of 10^9^ cm^−3^ throughout the whole period. The electron density radial distributions at the major and residual current peaks both in the positive and negative half cycles are depicted in Fig. 8(b–e). The electron density is plotted in the plane parallel to the electrodes, where the electron density reaches its maximum. All the electron density radial distributions present a concentric ring pattern but assume a center-advantage state overall. The electron density radial distribution at the residual current peak (*K*_1_ = 106.65 *μ*s) in the positive half cycle (see Fig. 8(b)) is very similar or the same as that at the residual current peak (*K*_3_ = 114.87 *μ*s) in the negative half cycle (see Fig. 8(d)). Figure 8(c,e) show that the electron density assumes the similar or same distributions at the two major current peaks (*K*_2_ = 108.63 *μ*s and *K*_4_ = 116.96 *μ*s) in the positive and negative half cycles respectively. It is found that the discharge has the similar or same spatiotemporal behavior in the two adjacent-half cycles and the nested complementary pattern disappears at the driving frequency of 60 kHz.

When the driving frequency varies from 21.5 to 60 kHz, period multiplication and chaos phenomena are distinguished from the temporal evolution of the discharge currents, which are not presented here. This behavior is attributed to the mismatch of different time scales that are important in electron production and loss^24^. The detailed discharge characteristics should be studied in the future.

Generally, it is difficult to realize a diffuse glow-like/Townsend-like DBD in argon at atmospheric pressure in experiments. Our simulation results predict the nested complementary pattern in the atmospheric-pressure argon DBD for some special conditions, under which the glow-like/Townsend like discharge is achieved. This pattern formation is explained by the charge transport regime. It is found that this unique behavior is mainly attributed to the fact that sufficient charged particles are trapped in the gas gap due to the last discharge and able to influence the subsequent discharge through the “spatial memory effect”. As for the argon DBD characterized by the nested complementary evolution of spatial discharge structure, there should be a residual discharge in glow mode and a subsequent discharge in Townsend mode before the major current pulse each half cycle. This allows the nested complementary pattern to only occur in a limited range of driving frequency.

## Methods

For this study, a two-dimensional (2D) self-consistent fluid model has been developed in an axisymmetric cylindrical coordinate (*r*−*z*) for the atmospheric-pressure argon DBD that has similar set-up to the one presented in ref. 25. The atmospheric-pressure argon discharge is generated and sustained between two parallel-plate circular electrodes (with the diameter of 6 mm), as depicted in Fig. 1. Each of the electrodes is covered by a dielectric barrier (with the thickness of 1 mm and relative permittivity of 7.5) and connected externally to a sinusoidal external voltage 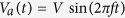. Here, *V* is the external voltage amplitude fixed at 1.8 kV and *f* the driving frequency of external voltage with its value varied from 12.5 to 60 kHz. The z-axis is directed from the lower barrier to the upper one with the upper surface of lower barrier designated as the origin.

In this model, the 2D simulation domain includes the two dielectric barriers and the discharge gap (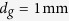). The gas temperature is assumed to be 300 K and the following species are considered: electrons, ions (Ar^+^), and metastable atoms (Ar^*^). Reactions including excitation, direct ionization, stepwise ionization, deexcitation, radiation, and electron-ion recombination in the bulk of gas gap are taken into account. All the reaction coefficients are referred to those used in refs. 26, 27, 28. The densities of all the particles are described by the continuity equation:


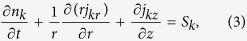


where *n*_*k*_, *j*_*k*_ and *S*_*k*_ represent number density, flux, and source term, respectively, for the *k*th kind of particles, and they are dependent on time and space. r and z are the radial and axial components respectively in the axisymmetric cylindrical coordinate. The fluxes 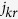 and 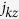 are deduced from the momentum equations:


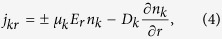



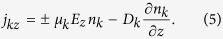


Here, the negative symbol in the first term on the right-hand side is taken for electrons and positive symbol for ions. *Er* and *E*_*Z*_ are the electric field along radial and axial direction respectively. *μ*_k_ is the mobility, which is set to be zero for the neutral particles, 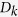 is the diffusion coefficient, and their values are taken from ref.^29^. In the source term 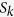, the reactions mentioned above are considered.

For the electron impact reactions, the rate coefficients are specified as functions of the mean electron energy, and the energy conservation equation electrons is included:


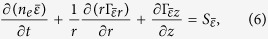


where 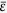 is the mean electron energy and *n*_*e*_ the electron density. The source term of this equation, 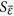, represents the energy gained in the electric field and the energy lost in collisions^28^. The electron energy fluxes in r and z direction are given by


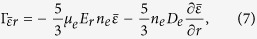



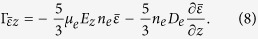


In addition, the Poisson equation is solved:


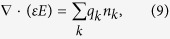


The r and z components of electric field *E*are satisfied with the current conservation equations:


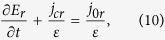



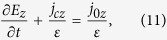


where *ε*is the permittivity, taking either *ε*_0_ in the gas gap or*ε*_0_*ε*_*B*_ in the dielectric barrier. 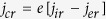 and 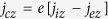 are the conduction current density along the r- and z-directions, respectively. The secondary electron emission from dielectric barriers through ion impact is taken into account by equation 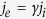, where the secondary electron emission coefficient *γ* is set to 0.01. The discharge current density along r-direction is zero and we only consider the value along z-direction^17^. Then the discharge current density *j*_0_ can be evaluated by integrating Eq. (11) from 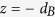 to 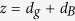:


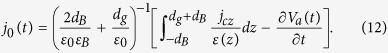


The set of equations above is numerically solved by using the semi-implicit Scharfetter-Gummel scheme on a non-uniform mesh of 2560 grids with the boundary areas (close to the dielectric barriers) meshed finer than the middle in the gas gap. To improve the efficiency of the simulation code, an adaptive time stepping is used with the initial time step of 10−13 s. It should be noted that all the simulation results discussed in this work are presented when the discharges are fully stabilized.

## Additional Information

**How to cite this article**: Jiang, W. *et al.* Prediction of nested complementary pattern in argon dielectric-barrier discharge at atmospheric pressure. *Sci. Rep.* **5**, 16391; doi: 10.1038/srep16391 (2015).
